# The Environmental Polymorphism Registry: A Unique Resource that Facilitates Translational Research of Environmental Disease

**DOI:** 10.1289/ehp.1003348

**Published:** 2011-06-09

**Authors:** Patricia C. Chulada, Enrikas Vainorius, Stavros Garantziotis, Lauranell H. Burch, Perry J. Blackshear, Darryl C. Zeldin

**Affiliations:** 1 Clinical Research Program, National Institute of Environmental Health Sciences, National Institutes of Health, Department of Health and Human Services, Research Triangle Park, North Carolina, USA; 2 Clinical Program, Integrated Laboratory Systems, Inc., Research Triangle Park, North Carolina, USA; 3 Laboratory of Molecular Genetics, and; 4 Laboratory of Signal Transduction, National Institute of Environmental Health Sciences, National Institutes of Health, Department of Health and Human Services, Research Triangle Park, North Carolina, USA

**Keywords:** DNA biorepository, environmental response gene, environmental risk assessment, genotypes, phenotype-by-genotype, polymorphisms, toxicity studies, translational research

## Abstract

Background: Dissecting complex disease has become more feasible because of the availability of large-scale DNA resources and advances in high-throughput genomic technology. Although these tools help scientists identify potential susceptibility loci, subjects with relevant genotypes are needed for clinical phenotyping and toxicity studies.

Objective: We have developed a resource of subjects and their DNA to use for translational research of environmental disease.

Methods: More than 15,000 individuals of diverse sex, age, race, and ethnicity were recruited from North Carolina. DNA was isolated from their blood and coded with personal identification numbers linked to their identities. This linked resource of subjects and their DNA—the Environmental Polymorphism Registry (EPR)—allows scientists to screen for individuals with genotypes of interest and invite them to participate in follow-up studies.

Discussion: The EPR is a phenotype-by-genotype resource designed to facilitate translational studies of environmental disease. Based on their genotypes, subjects are invited to participate at all levels of research, from basic laboratory *ex vivo* cell phenotyping experiments that require viable tissue to *in vivo* observational studies and clinical trials. Here we report on progress of the EPR since 2008. We also describe a major effort at the National Institute of Environmental Health Sciences (NIEHS) to investigate susceptibility loci in 87 environmental response genes and gene × environment interactions using EPR resources.

Conclusion: The EPR is a unique and novel resource and is ideal for genotype-driven translational research of environmental disease. We expect that it will serve as a model for future resources. Such tools help scientists attain their ultimate goals: to identify at-risk populations and develop strategies for preventing and treating human disease.

Translational research of complex human disease is dependent on the availability of well-annotated genetic materials for identifying susceptibility loci, viable biospecimens for functional single-nucleotide polymorphism (SNP) assays, and research subjects for *in vivo* phenotyping and toxicity studies. A wide spectrum of human tissue repositories are available today for genomic and proteomic research. These repositories, in combination with high-throughput genotyping and bioinformatics, have greatly advanced the understanding of genetic alterations and biological pathways that influence human disease. However, identifying appropriate human research subjects with genotypes of interest for follow-up studies remains problematic. With this in mind, we have created a unique resource to facilitate translational research of environmental disease. This is a large-scale phenotype-by-genotype registry consisting of > 15,000 individuals of diverse sex, age, race, and ethnicity, named the Environmental Polymorphisms Registry (EPR) [Chulada et al. 2008; National Institute of Environmental Health Sciences (NIEHS) 2011].

The EPR is a linked DNA biorepository. DNA is isolated from blood samples donated by the subjects and linked to their identities through a personal identification number. Compared with anonymous biorepositories, linked resources such as the EPR are more useful in translational research. With the former, information on the donors’ identities is permanently destroyed, precluding follow-up of the same individuals known to have functionally relevant genotypes. With linked repositories such as the EPR, subjects with genotypes of interest can be asked to participate in all phases of translational research, from basic laboratory *ex vivo* cell phenotyping studies requiring viable tissue to comprehensive *in vivo* clinical phenotyping and/or toxicity studies. Other types of studies are possible, including cohort studies of disease risk, and interventional and personalized medicine trials.

The EPR has been described previously (Chulada et al. 2008; NIEHS 2011). Here we report on our progress in establishing the EPR since 2008 and its use in several follow-up studies. We also describe a major project to investigate potential susceptibility loci in 87 environmental response genes using EPR subjects. This project (the EPR Consortium Project) is being conducted in two phases. In the first phase, EPR subjects are screened for potentially significant loci using high-throughput genotyping methods. In the second phase, subgroups with shared genotypes are asked to participate in various follow-up studies to examine cellular and clinical phenotypic differences. Many of the studies involve exposing subjects (or cells) to environmental stimuli and examining gene × environment interactions. The purpose of this commentary is to illustrate how linked resources like the EPR facilitate genotype-driven translational research of environmental disease, and help scientists identify at-risk populations and develop strategies for preventing and treating disease.

## EPR Progress since 2008

The EPR was designed to facilitate genotype-driven research. During enrollment, subjects consent to anonymous phase 1 genetic screening and to being reidentified and called back for phase 2 phenotyping studies on the basis of their genotypes. Phase 2 phenotyping studies are voluntary; subjects can choose to take part in some studies and not in others. No subjects are reidentified for phase 2 studies until a protocol is developed and reviewed by an advisory oversight committee (the EPR Steering Committee), scientific review committee, and institutional review board (IRB). EPR methods, human subjects protection measures, advisory oversight, and other aspects have been described in detail previously (Chulada et al. 2008).

EPR recruitment began in 2005 and initially encompassed counties surrounding Research Triangle Park, North Carolina. The goal was to enroll 3,000–5,000 subjects per year until 20,000 subjects were recruited. To meet this goal, we expanded recruitment to outlying counties in 2007; by 2009 we had enrolled almost 15,000 subjects. Thereafter, the rate of recruitment decreased because of budgetary constraints.

**Figure 1:**
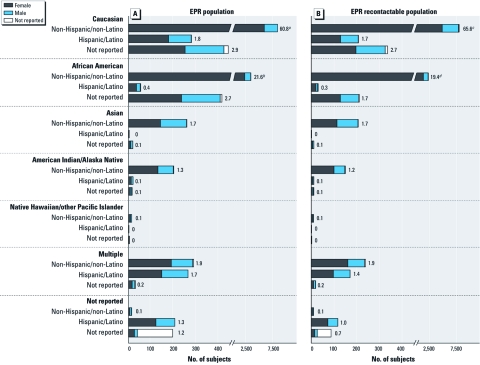
Demographics of the entire EPR population (*A*) and of the recontactable EPR population (*B*). Numbers beside bars represent percentage of the population. ***^a^***37.5% female, 22.6% male, and 0.7% not reported. ***^b^***14.2% female, 7.2% male, and 0.2% not reported. ***^c^***40.8% female, 24.1% male, and 0.7% not reported. ***^d^***13.4% female, 5.9% male, and 0.1% not reported.

At the time of our first report, the EPR consisted of 7,788 subjects (Chulada et al. 2008). Today, there are twice that number (*n* = 15,376 subjects of diverse sex, age, race, and ethnicity). These include 65.5% Caucasians, 24.7% African Americans, and smaller percentages of other races (Figure 1A). The racial makeup is representative of North Carolina based on 2000 Census data (U.S. Census Bureau 2011) (African Americans are somewhat overrepresented). A significant proportion of EPR subjects (5.2%) are Hispanic or Latino (Figure 1A), and most (61.6%) are female (Table 1). Most subjects are young (18–40 years of age) or middle-age (41–60 years) adults (Table 1). This makes them ideal for studying adult-onset disease.

The EPR is a long-term registry; subjects have agreed to be recontacted for follow-up studies for 25 years after enrollment. Therefore, we maintain current contact information on as many subjects as possible using a combination of annual mailings, telephone calls, and tracing (Chulada et al. 2008). EPR subjects are considered active (for phase 2 studies) if they are newly recruited (within a year) or have been successfully recontacted within the past year. Recontacting efforts have been successful; 80.5% (*n* = 12,375) of the EPR population remains active (recontactable) since recruitment began in 2005 (Figure 1B).

**Figure 2:**
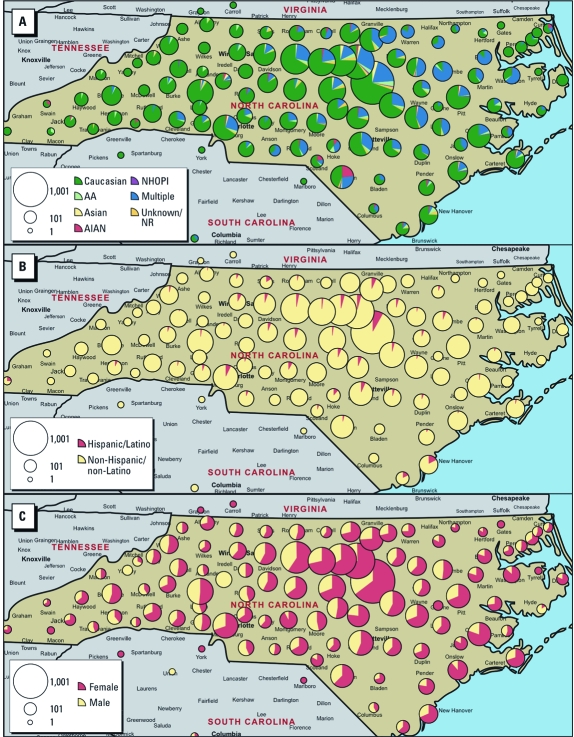
Distribution of the recontactable EPR population in North Carolina by race (*A*), ethnicity (*B*), and sex (*C*). The size of each pie represents an estimate of the number of EPR subjects living in the region under the pie. Pie slices represent the proportions of subjects of different races (*A*), ethnicity (*B*), and sex (*C*). Abbreviations: AA, African American; AIAN, American Indian/Alaska Native; NHOPI, Native Hawaiian/other Pacific Islander; NR, not reported.

Most EPR subjects reside in the Research Triangle Park region and can readily travel to the NIEHS or other research centers for follow-up studies. Figure 2 depicts the distribution of subjects in North Carolina by race, ethnicity, and sex. About 8.1% of active subjects live out of state (most in Virginia and South Carolina, which border North Carolina), and 0.1% live outside the United States.

## EPR Consortium Project

*Phase 1: genetic screening.* The EPR Consortium Project is a large translational research project initiated at the NIEHS by a multidisciplinary team of basic scientists, geneticists, toxicologists, clinicians, and biostatisticians (the EPR Consortium). Consortium members have selected 87 genes for study that, based on cell culture, animal, and/or human studies, are candidate genes for asthma, atherosclerosis, cancer, autoimmune disease, aging, and other conditions. Most are environmental response genes that work in concert with environmental exposures to elicit a phenotype. Examples include cytochromes P450 (*CYP2J2*, *CYP2C8*, *CYP2C9*), which are involved in xenobiotic or drug metabolism, and *AhR* (aryl hydrocarbon receptor), *ARNT* (AhR nuclear translocator), and *AhRR* (AhR repressor), which mediate the effects of polycyclic aromatic hydrocarbons and other endocrine- and immune-disrupting xenobiotics.

From the 87 genes, we identified 717 SNPs that were predicted to alter protein sequence and/or function, are in evolutionarily conserved regulatory regions, or tag European and African ancestral haplotypes. About 70% of these SNPs can be found in dbSNP [National Center for Biotechnology Information (NCBI) 2010b] and HapMap databases (NCBI 2010a) and were selected using SNPselector (Xu et al. 2005) or based on *a priori* functional significance. The other 30% of SNPs are novel and potentially significant based on research conducted by individual consortium members. An additional 51 sex and ancestral informative markers (AIMs) are being genotyped and will be compared with self-reported sex and race for quality control. The AIMs also will be used to measure admixture in the population and to adjust for population stratification.

In this first round of genotyping, 4,000 subjects are being screened using custom high-throughput 384-plex Illumina arrays (Illumina, Inc., San Diego, CA). The 4,000 consists of approximately 500 subjects of Hispanic or Latino ethnicity and equal numbers of males and females of African and European ancestry. Important aims of phase 1 genotyping are to assess genotype frequency in the EPR population stratified by race, ethnicity, and sex, inform phase 2 study design, and identify appropriate subjects for follow-up studies. We expect that most (but not all) genotyping studies will lead to follow-up phenotyping studies, and in these situations we screen only active subjects who can be readily recontacted. Nonactive EPR subjects can be used in genotyping projects where follow-up is not important, for example, in simple assessments of SNP prevalence rates or in approximation of haplotypes using statistical methods (Stephens et al. 2001).

*Phase 2: phenotyping.* Based on phase 1 results, subgroups of EPR subjects with shared genotypes are invited to participate in various phase 2 studies. The studies vary in hypotheses, and their design depends on minor allele frequencies, population stratification, and gene penetrance. Cell phenotyping studies have been the most common type of follow-up study proposed to date, as we expected in early-phase EPR research. Subjects with genotypes of interest are invited to donate viable tissue for basic laboratory experiments aimed at characterizing some molecular or functional attribute of the genotype. Here statistical power depends on the allele frequency and magnitude of the biochemical or molecular effect, and small numbers of subjects are usually adequate. Higher levels of follow-up studies have been proposed and include observational or interventional clinical trials, epidemiological surveys, and cohort studies of disease risk. Four follow-up studies are described below to illustrate the usefulness of the EPR in translational research. The first three have been approved by the NIEHS IRB and are under way; the fourth is under review.

As described above, cell phenotyping studies are a common use of EPR resources. In the first example, we screened subjects for SNPs in p53 response elements of *p53* downstream genes (*FLT1*, *TLR8*, *RRM1*, *MDM2*). During a follow-up study, these subjects were asked to donate blood for viable lymphocytes to test the potential of the SNPs to alter cell function, p53 promoter occupancy, and transactivation of downstream genes by p53 tumor protein (Bond et al. 2004; Menendez et al. 2006; Murphy 2006; Tomso et al. 2005). We treated the cells *ex vivo* to induce p53-mediated stress and DNA damage and examined them for gene expression by microarray technology.

In the second example, viable mononuclear cells were isolated from the blood of subjects with SNPs in *ApoE*, *ABCA1*, and other genes that regulate cholesterol trafficking and immune response (Mahley and Rall 2000; Singaraja et al. 2003); these cells were then used to test the potential of SNPs to alter inflammatory response following *ex vivo* bacterial lipopolysaccharide challenge. In both the first and second examples, we also recruited appropriate genetic controls from the EPR and matched these subjects for sex, race, and ethnicity to subjects with the minor alleles.

The EPR can provide adequate numbers of subjects for highly powered cell phenotyping studies such as those described above. To test the null hypothesis (no differences between genotypes) using *t*-tests, in the first example 10 subjects were needed with each genotype to detect a 1.4-fold change in gene expression (90% power). In the second example, 9 subjects with each genotype were needed to detect a 1.3-fold change in cytokine (tumor necrosis factor-α) levels (85% power). These calculations assume a significance level of 0.05.

In the third example, we used EPR resources at different levels of research, starting with basic cell phenotyping experiments that led to comprehensive clinical observational studies. First, we isolated viable mononuclear cells from EPR subjects harboring potentially functional SNPs in *hGR* (human glucocorticoid receptor) (Jewell and Cidlowski 2007; Schaaf and Cidlowski 2002). The cells were exposed *ex vivo* to glucocorticoids and examined for immune function and gene expression. At the next level, subjects with impaired cellular immune function underwent modified dexamethasone suppression tests to examine the potential of SNPs to alter steroid responsiveness. We also examined subjects for risk factors (body mass index, hip:waist ratio, and blood levels of cortisol, lipids, glucose, insulin, and other metabolites), family history with emphasis on inflammatory and metabolic disease (Manenschijn et al. 2009), and stress. The goals were to examine how cells, organs, and humans respond to physiological and environmental stressors and how polymorphisms in *hGR* affect those responses.

Glucocorticoids that elicit responses through *hGR* regulate numerous homeostatic functions (glucose homeostasis, protein and lipid metabolism, skeletal growth, connective tissue metabolism, respiratory function, immune surveillance, and behavior) (Ren and Cidlowski 2005). Glucocorticoids are also among the most prescribed drugs in the world and are a primary treatment for inflammatory and immune disease (asthma, arthritis, inflammatory bowel disease). Chronic elevation of glucocorticoids from prolonged stress and/or chronic therapeutic administration can have detrimental effects on human health. Therefore, by identifying significant *hGR* polymorphisms and understanding how they affect glucocorticoid responsiveness, we can identify populations at risk for these conditions and/or predict how others might respond to glucocorticoid therapy. Next levels in this line of research might include a cohort study or personalized medicine trial, both possible using EPR resources.

The fourth example is a clinical toxicity study designed to examine gene × environment interactions. EPR subjects with functional SNPs in *CD44* (receptor for hyaluronic acid) and *I*α*I* (inter-α-inhibitor) will be exposed to ozone via inhalation and examined for bronchoconstrictive responses to inhaled methacholine. In addition, associations between the SNPs and inflammatory and immune markers will be examined in peripheral white blood cells and alveolar macrophages collected from the same subjects. In animals and humans, both genes have been shown to have roles in ozone-induced airway hyperreactivity (Garantziotis et al. 2009) and/or inflammatory responses in alveolar macrophages (McKee et al. 1996). The ultimate goal is to identify populations at risk for lung inflammation after ozone exposures.

## Discussion

Translational research of complex disease involves identifying underlying susceptibility loci and the environmental factors that affect development and/or progression of disease, and then applying this information to clinical strategies for predicting, preventing, diagnosing, and/or treating disease. This process is complicated by the fact that complex disease involves multiple genes that individually have small phenotypic effects, and the effects of individual genes are predicated on environmental triggers and complex pathways. Translational research therefore begins by characterizing the phenotypic effect of a single locus for some molecular or functional change. As new findings emerge, progressively higher levels of study evolve that require *in vivo* clinical investigation of individuals with the relevant genotypes and their responses (or resulting phenotypes) to environmental triggers. Having the appropriate tools (DNA, viable tissue, subjects with genotypes of interest) can facilitate the translational research process, and that was our purpose for developing the EPR.

The EPR is a unique and novel registry of subjects and their DNA and was designed to facilitate genotype-driven research of environmental disease. The EPR Consortium was assembled to identify important environmental response genes and design studies to test hypotheses concerning gene × environment interactions using EPR resources. In deciding which projects to support, preference is given to those with potential for identifying at-risk populations or where the results are applied to clinical practice. For example, in the *hGR* study described above, the results will inform scientists about populations at risk for multiple conditions after stress. Future personalized clinical trials using EPR subjects might help clinicians identify patients for whom glucocorticoid therapy might be more effective or toxic. The *CD44*/*I*α*I* study described above will identify populations at risk for inflammatory lung disease after ozone exposure. Preventative strategies might then be developed for people living in high-ozone areas who carry *CD44* and *I*α*I* SNPs, or individuals who develop inflammatory lung disease might be studied for targeted therapies.

Other types of follow-up studies have been conducted (or proposed) using EPR resources in addition to those described here. These include gene modifier and pharmacokinetic studies, epidemiological surveys, interventional trials, and ethics/opinion surveys. Although the EPR is suitable for many types of studies, we emphasize that it is not intended for large association or all-inclusive epidemiological studies. Instead, the EPR is intended to answer refined phenotyping questions, with narrowly defined hypotheses and specific, measurable end points.

*Benefits of EPR.* The diversity of the EPR population is a major benefit. This allows scientists to stratify studies by sex, age, race, and ethnicity. The EPR has a large minority population that consists of 24.7% African Americans and 5.3% Hispanics or Latinos. Minorities were targeted in the *hGR* study because of a lack of relevant data in these populations (Chung et al. 2009; Hawkins et al. 2004). The EPR is monitored for racial diversity and recruitment is targeted toward underrepresented groups as needed.

Another benefit is the transparency of the informed consent process. Subjects are informed verbally and in writing, using plain language, of the potential uses of their samples and the protections in place to protect their privacy. They are made aware that although their DNA is coded during phase 1 genetic screening, their personal information will be shared with scientists for phase 2 follow-up studies. They are told to expect to be recontacted each year to update their personal information, how often they might be contacted for follow-up studies, what participation in follow-up studies might entail, and that participation is voluntary at the time asked. This reassures subjects regarding uncertainties associated with future uses of their samples and provides them with a solid understanding of study logistics and goals. Measured EPR response rates at study drives (rate of people who sign the written consent form following verbal summation) are typically > 95%.

A unique feature of the EPR is that attrition is minimized by maintaining continual long-term contact with subjects. To date, 80.5% of the EPR population is active, meaning they have updated their contact information within the past year and are available for follow-up studies. Considering that recruitment began in 2005, we regard this as a success. Furthermore, yearly rates of attrition have decreased over time as our methods for tracking subjects have become more sophisticated. Subjects are now asked to provide alternate contacts and will soon be recontacted by e-mail in addition to the mailings by post and phone calls. Subjects also receive biannual newsletters that discuss EPR progress, events, and follow-up studies [for a copy of the first EPR newsletter, see Supplemental Material (http://dx.doi.org/10.1289/ehp.1003348)].

*Limitations of the EPR.* A limitation of the EPR is a lack of associated health, family history, and other types of data. This will be remedied soon: Over the next year we will survey EPR subjects about their health status and family history. The survey was designed with input from consortium members and other scientists who have a stake in EPR research. In addition, home addresses of EPR subjects will be modeled spatially using geospatial information systems (GIS) technology. This project is being conducted in collaboration with M.L. Miranda (Nicholas School of the Environment and Earth Sciences, Duke University) and will allow us to characterize subjects (based on where they live) for demographics, culture, health outcomes, environmental quality, chemical exposures, and other factors. GIS modeling allows us to visualize data in ways that might reveal relationships, patterns, and trends and can be used to inform follow-up study design. Miranda and colleagues have previously used GIS modeling to predict lead exposure risk levels in North Carolina children (Miranda et al. 2002) and to identify first Gulf War veterans at risk for amyotrophic lateral sclerosis based on toxic exposures in Iraq (Miranda et al. 2008).

A potential limitation of the EPR is the sampling method used. Subjects were recruited in clusters throughout North Carolina as a convenience sample, which has advantages and disadvantages. The major advantage is that subjects can be ascertained quickly and inexpensively. This was a consideration in developing a strategy to recruit 20,000 subjects within a relatively short time. A disadvantage is the potential for sampling bias. The basis of the EPR population is geography (state of North Carolina), and although the EPR is representative of North Carolina in terms of race and ethnicity, its representativeness in other areas (socioeconomic, health status, other) is not known. Although we might conduct limited analyses of the EPR as a population-based sample, it will primarily be used for smaller functional and phenotyping studies where subjects are selected based on genotype. Therefore, we expect that sampling bias will have only small effects on individual follow-up studies.

Nonresponse bias might be more problematic. EPR subjects are recruited from the general populace via outpatient health care clinics, health fairs, community groups and gatherings, corporate study drives, and the Internet. These types of venues might bias the population toward more health-conscious individuals and/or fewer individuals with disease-causing alleles. This could be compounded further by self-selection at study drives. Whether nonresponse bias will affect particular follow-up studies depends on the hypothesis and subject recruitment criteria.

## Conclusion

Phenotype is commonly used as the basis for selecting subjects for biomedical and epidemiological research of complex disease. This approach is sometimes problematic because of broad and heterogeneous phenotypes, poor phenotyping criteria, heterogeneous populations, selection bias, and a host of other issues. Advances in genomic technology combined with large-scale genetic repositories are improving the ways complex diseases are studied, and have shifted the basis of subject selection toward genotype. Resources such as the EPR are valuable tools in genotype-driven research and can be used to characterize variants “from bench to bedside” as they are discovered from epidemiological research.

The EPR is novel and unique. As a subject registry and linked DNA biorepository, it offers scientists advantages over anonymous biorepositories. It provides scientists not only DNA to identify potentially significant genetic variants but also a readily recontactable and diverse population for observational and toxicity studies and clinical trials. Once EPR subjects have been better characterized for health status, exposures, lifestyles, and other factors, scientists can generate more focused study hypotheses, design better follow-up studies, and select appropriate subpopulations to study.

The EPR builds upon the Environmental Genome Project (EGP) first launched at the NIEHS in 1997 to address the role of genetic variation in response to environmental exposure in large populations (Guengerich 1998). EGP goals were to identify polymorphisms in environmental response genes, study the functional implications of the polymorphisms, and associate them with various diseases in large population-based studies. The goals of the EPR are similar, but the EPR provides scientists with better resources for meeting these goals and allows scientists to take a flexible, stepwise translational research approach into complex disease mechanisms. Knowledge of these mechanisms using EPR resources will lead to new preventative, diagnostic, and/or therapeutic interventions that can significantly improve the public’s health.

## Supplemental Material

(900 KB) PDF
