# Supplementary material for: The Environmental Polymorphism Registry: A Unique Resource that Facilitates Translational Research of Environmental Disease
Source: Environ Health Perspect. 2011 Jun 9;119(11):1523–7. doi: 10.1289/ehp.1003348 (PMC3226495; doi:10.1289/ehp.1003348)
Supplement: (900 KB) PDF [file ehp.1003348.s001.pdf]

Supplemental Material

Title: The Environmental Polymorphism Registry – a Unique Resource that Facilitates Translational Research of Environmental Disease

Authors: Patricia C. Chulada,[1] Enrikas Vainorius,[2] Stavros Garantziotis,[1] Lauranell H. Burch,[3] Perry J. Blackshear,[4] Darryl C. Zeldin[1]

Institutions where work was performed: National Institute of Environmental Health Sciences, Research Triangle Park, NC 27709; University of North Carolina, Chapel Hill, NC 27514

Authors affiliations: [1]Clinical Research Program, National Institute of Environmental Health Sciences, Research Triangle Park, North Carolina, USA; [2]Clinical Program, Integrated Laboratory Systems, Inc., Research Triangle Park, North Carolina, USA; [3]Laboratory of Molecular Genetics, National Institute of Environmental Health Sciences, Research Triangle Park, North Carolina, USA; [4]Laboratory of Signal Transduction, National Institute of Environmental Health Sciences, Research Triangle Park, North Carolina, USA.

Corresponding author:

Patricia C. Chulada, National Institute of Environmental Health Sciences, 111 T.W. Alexander Drive, Research Triangle Park, NC 27709, Phone: 919-541-7736, Fax: 919-541-3715, E-mail: Chulada@niehs.nih.gov.

Updating Your Contact Information

The success of the EPR depends on us being able to contact you for future studies, such as those described in this newsletter. Therefore, it is very important that we have your current address, telephone numbers, and email. This is the reason we send you all those letters each year asking you to confirm or correct your contact information. When you receive these letters, please follow the instructions and return the enclosed update card to us by mail, even if your contact information has not changed. If you prefer, you can also call (1-866-809-1261) or email (epr@sra.com) us with this information.

Have Questions?

If you have questions about the EPR, or would like to contact us for any reason, we would love to hear from you!

Study Contacts:

Pat Chulada, Ph.D., M.H.S.
Telephone: (919) 541-7736
E-mail: chulada@niehs.nih.gov

Beverly A. Warden, P.M.P., Ph.D, M.P.H.
Telephone: (919) 313-7558
E-mail: beverly_warden@sra.com

EPR Toll-Free Hotline: 1-866-809-1261
E-mail: epr@sra.com
http://www.niehs.nih.gov/epr

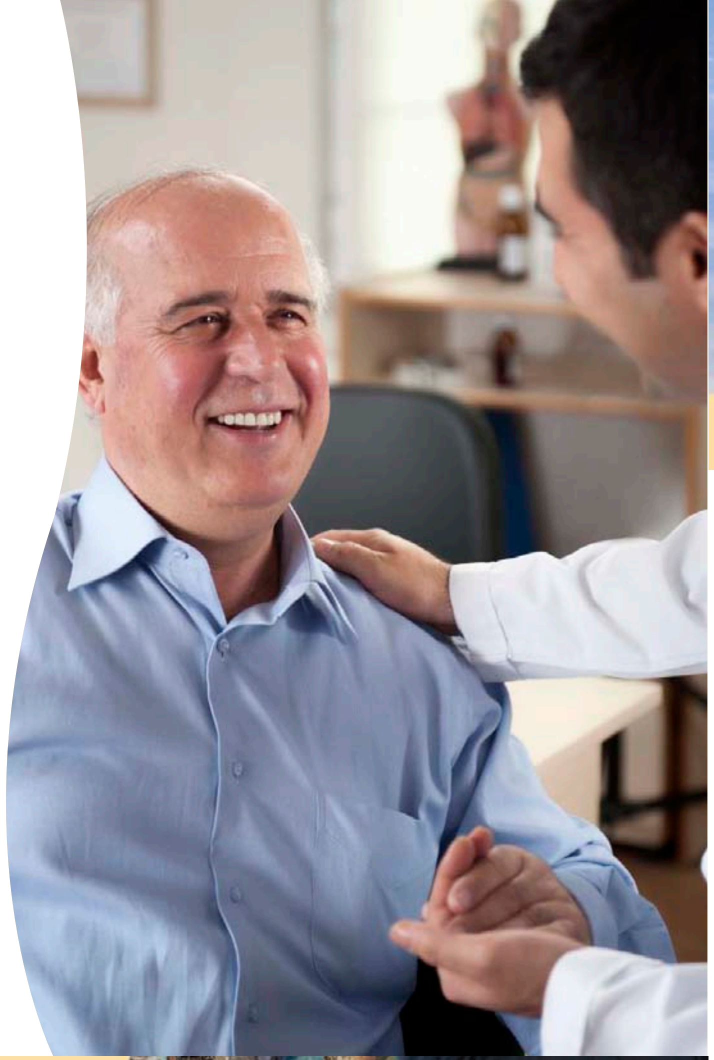

115

OFFICIAL BUSINESS

Research Triangle Park, NC 27709-2233
U.S. PO BOX 12233
National Institute of Environmental Health Sciences
National Institutes of Health
Department of Health and Human Services

Environmental Polymorphisms Registry NEWSLETTER

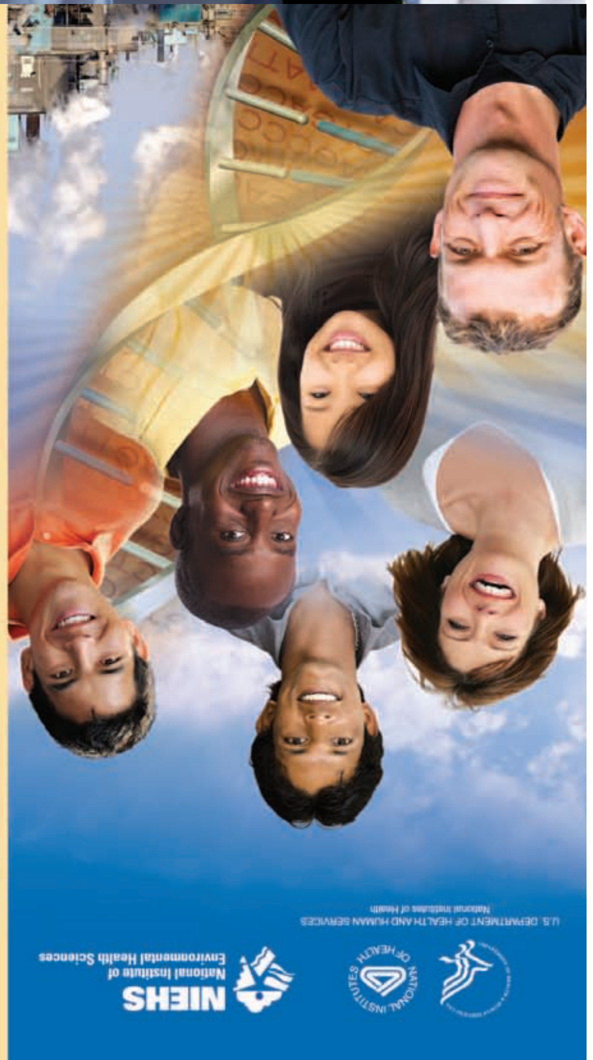

NIEHS
National Institute of Environmental Health Sciences

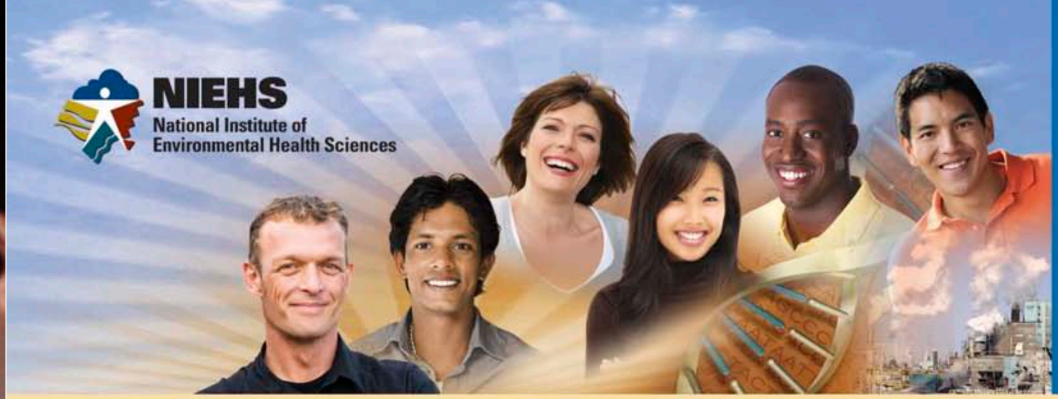

Environmental Polymorphisms Registry NEWSLETTER

http://www.niehs.nih.gov/epr

June 2011 Issue

Welcome and Thank You!

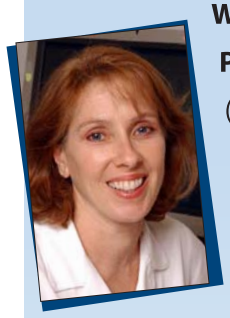

We at the Environmental Polymorphisms Registry (EPR) would like to thank all our participants for their generous support and continued interest in this project. We are also pleased to introduce to you our first biannual newsletter to let you know about EPR events.

The EPR has been a great success. We began recruiting in 2006 and now have over 15,000 participants! We owe this success to you, and look forward to your continued participation. We also have several new and ongoing EPR studies to tell you about, so read on. But first, "What in the world are polymorphisms?"

Sincerely,

Patricia C. Chulada

Pat Chulada, Ph.D., M.H.S.
EPR Director

What are Polymorphisms?

Polymorphisms are nothing more than differences in our genetic code or DNA, which make up our genes. Everyone has polymorphisms in their genes – they are what make us different from one another, giving some people brown hair and others blue eyes. Each of us has millions of polymorphisms in our genetic make-up, the vast majority of which have no known function or significance. Polymorphisms in certain genes, however, can affect the efficiency of those genes, and can affect our health.

In the EPR, scientists study a special group of genes known as the "environmental response genes." These genes work in combination with environmental factors and our lifestyles to increase (or sometimes decrease) our chances for developing conditions like cardiovascular disease, cancer, asthma, and many others. Almost all disease is caused by a combination of genetic, environmental, and lifestyle factors.

Why is the EPR important?

The EPR was developed as a research resource to help scientists study conditions like those mentioned above. Our goals are to recruit up to 20,000 participants from North Carolina and collect a blood sample from them for DNA extraction. The DNAs are placed in a tissue bank also known as a biobank. Scientists

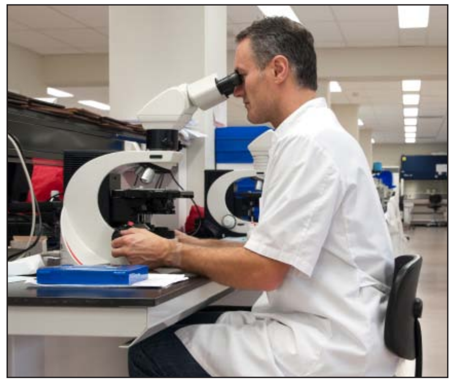

can screen DNAs from the biobank for polymorphisms in specific "environmental response genes." The polymorphisms might or might not affect the efficiency of the genes and that is what the scientists are trying to find out.

There are many DNA biobanks available for scientists to use. The EPR is a unique biobank compared with most others. With the EPR, the identities and contact information of our participants are "linked" back to their DNAs through a coded identification number. This gives scientists the ability to recontact EPR participants and ask them to volunteer for follow-up studies based on their specific polymorphisms. With other DNA biobanks, the personal identifiers of the DNA donors are destroyed. Therefore, scientists can only use the DNAs to select polymorphisms for study

U.S. DEPARTMENT OF HEALTH AND HUMAN SERVICES
National Institutes of Health

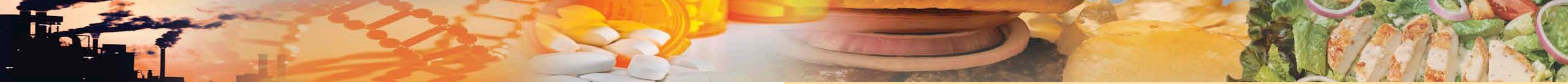

CONTINUED FROM PAGE ONE

but can't ask the donors of those DNAs to participate in future studies. Instead, they have to rescreen new populations for participants, and that is both time consuming and expensive.

As you have noticed with the EPR, we go through great lengths to maintain up-to-date contact information on all our participants. This is very important as the success of the EPR and its future studies depends on us being able to contact you. ■

New EPR Studies

EPR Gene Project – Phase 1

The EPR Gene Project (Phase 1) is a large study currently underway at the National Institute of Environmental Health Sciences (NIEHS) that encompasses a wide variety of genes and conditions. DNAs from 4,000 EPR participants of diverse racial and ethnic backgrounds are being screened for over 800 polymorphisms in 87 different "environmental response genes." Some of the genes are metabolizing genes – they break down chemicals in our bodies to their non-toxic forms which are more readily excreted from our bodies. Other genes being studied in the EPR Gene Project include inflammatory and immune response genes.

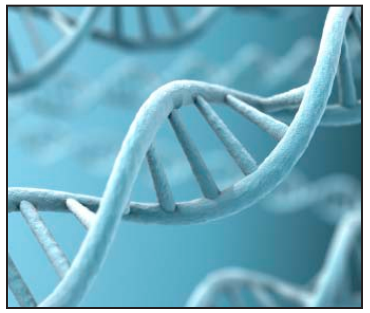

Inflammation is our body's first response to harmful stimuli such as pathogens (viruses, bacteria), damaged cells in our bodies, or irritants. Inflammatory genes work in a nonspecific manner to remove the stimuli or damaged cells and begin the healing process. Immunity is our body's secondary response to harmful stimuli. Immune response genes recognize specific proteins (antigens) in allergens (substances like pollen, animal dander, dust, mold, and grasses) and mount very specialized responses to these proteins. In the last few years, both inflammatory and immune response genes have been implicated in a wide variety of conditions and are an active area of research today.

New technologies are being used in the EPR Gene Project that allows us to screen a high number of DNA samples for numerous polymorphisms quickly and inexpensively. Once all 87 genes have been analyzed, we expect multiple follow-up studies to be conducted in which EPR participants with polymorphisms in these genes will be asked to volunteer. ■

Health Questionnaire

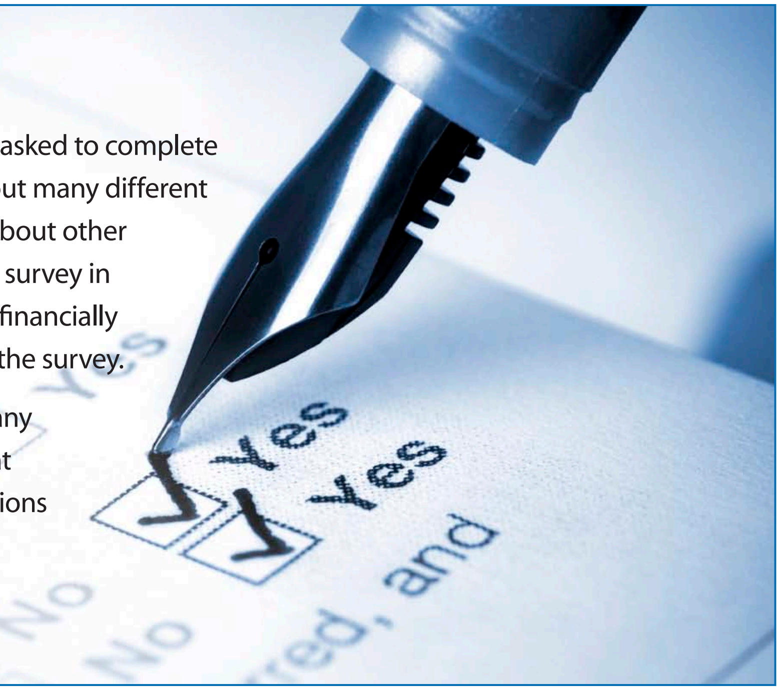

Over the next year or so, all EPR participants will be asked to complete a health survey. In this survey, you will be asked about many different conditions, your family's health, your lifestyle, and about other information. You will receive information about this survey in the mail over the next few months. You will also be financially compensated for the time it takes you to complete the survey.

The success of this survey depends on having as many participants complete it as possible, so we hope that you will find the time to do so. If you have any questions about this survey, please contact Andrea Zombeck at andrea_zombeck@sra.com or 919-313-7618.

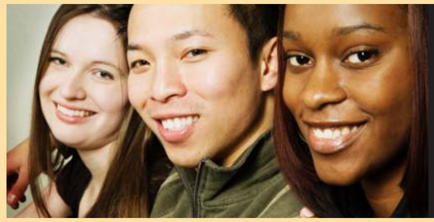

Follow-Up Studies

Geographic Information Systems Study

This is an exciting new EPR study involving state of the art Geographic Information Systems (GIS) technology. The study is being conducted by EPR Director Pat Chulada in collaboration with Duke scientist Marie Lynn Miranda. EPR participants will be geographically mapped (assigned latitude and longitude coordinates) based on where they live. The coordinates will then be plugged into various environmental quality databases to determine their exposures. For example, GIS coordinates will be used to search the Air Quality Systems database developed by the U.S. EPA. This will provide us information on air pollutant levels (carbon monoxide, particulate matter, ozone, and others) for the locations where EPR participants live. We can also track levels of pollutants over time (retrospectively and prospectively) to quantify exposures. These data will then be analyzed in conjunction with health data collected from EPR participants *(see Health Questionnaire)* as well as with their genetic profiles *(see EPR Gene Project – Phase 1)*.

We are very excited about this study and our collaboration with Duke University. Using GIS technology in this manner, we can visualize data in various ways that reveal relationships and patterns between exposures, health, and genes. Stay tuned for our next newsletter to learn how GIS technology is being used to examine environmental and genetic risk factors for cardiovascular disease. ■

Role of Glucocorticoids in Cardiovascular and Metabolic Disease

Inflammatory and immune responses (described above) are important protective mechanisms when our bodies are exposed to toxic stimuli. However, when the responses persist for long periods of time, they can actually damage our bodies. Glucocorticoids (GCs) are hormones that protect us against our own responses by turning down inflammatory and immune responses. GCs are also given therapeutically for this same reason.

GCs exert their anti-inflammatory and anti-immune effects by binding to proteins called glucocorticoid receptors. In this EPR study, scientists John Cidlowski and Chris Jewell at the NIEHS are studying whether DNA polymorphisms in glucocorticoid receptor genes alter the binding of GCs, and subsequently alter our ability to turn down inflammatory and immune responses. Their ultimate goal is to determine whether polymorphisms in these genes increase our risks for cardiovascular or metabolic (diabetes, high blood lipids, obesity) disease following exposure to inflammatory and immune stimuli.

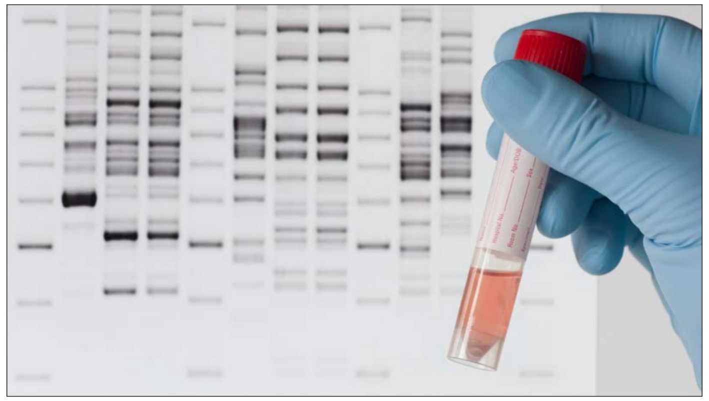

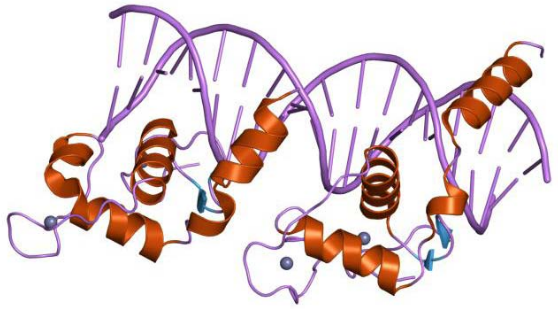

In this study, participants with polymorphisms in glucocorticoid receptor genes will be given low doses of GCs. Blood will be collected before and after taking the GCs and measured for inflammatory and immune factors. ■
